# Effect of Membrane Perforations With and Without Collagen Membrane Coverage in Vertical Ridge Augmentation: An Experimental Pilot In Vivo Study

**DOI:** 10.1111/clr.70157

**Published:** 2026-07-19

**Authors:** Istvan A. Urban, Sándor Farkasdi, Muhammad H. A. Saleh, Monica Lopez, Fernando Muñoz, Gabor Varga, Mauricio G. Araujo, Fabio Vignoletti

**Affiliations:** ^1^ Urban Regeneration Institute Budapest Hungary; ^2^ Department of Periodontics and Oral Medicine University of Michigan School of Dentistry Ann Arbor Michigan USA; ^3^ Department of Oral Biology, Faculty of Dentistry Semmelweis University Budapest Hungary; ^4^ Department of Veterinary Clinical Sciences Universidad de De Santiago de Compostela Lugo Spain; ^5^ Centre for Translational Medicine Semmelweis University Budapest Hungary; ^6^ Department of Oral Biology Semmelweis University Budapest Hungary; ^7^ State University of Maringá Maringá Brazil; ^8^ Private Practice in Dental Implants and Periodontics Rio de Janeiro Brazil; ^9^ Department of Periodontology Complutense University of Madrid Madrid Spain; ^10^ Thinking Perio Research, Studio Dentistico Vignoletti Verona Italy

**Keywords:** alveolar ridge augmentation, angiogenesis, animals, dental implants

## Abstract

**Objectives:**

Given the proposed bioactive role of collagen membranes, we aimed to evaluate whether the perforation size of titanium‐reinforced dPTFE membranes, with or without an additional collagen membrane, influences outcomes of vertical alveolar ridge augmentation (VARA).

**Materials and Methods:**

In 12 beagle dogs (24 sites), standardized bilateral chronic mandibular ridge defects (8 × 30 mm) were created and healed for 12 weeks. Sites were randomized (1:1:1) to VARA using porcine xenograft covered with either: (i) non‐perforated titanium‐reinforced dPTFE (NON‐PERF), (ii) perforated titanium‐reinforced dPTFE (PERF), or (iii) perforated titanium‐reinforced dPTFE covered by a collagen membrane (PERF+CM). After 16 weeks, specimens underwent micro‐CT, histology/histomorphometry, vessel quantification, and immunohistochemistry (OCN, ALP, VEGF, TRAP). Continuous outcomes were summarized as medians and compared using the Kruskal–Wallis test with Dunn's post hoc testing when applicable; inflammatory cell scores were analyzed using ordinal logistic regression (*α* = 0.05).

**Results:**

Of 24 treated sites, four had complications and were excluded. All remaining sites achieved around 60% new bone area with no significant intergroup differences (new bone *p* = 0.5047; soft tissue *p* = 0.2994; residual graft *p* = 0.9689). Linear vertical gain was comparable (medians 2.33, 2.87, 2.74 mm; *p* = 0.7119). Micro‐CT outcomes did not differ (e.g., BV/TV, *p* = 0.2739). Ordinal models did not show significant group differences in inflammatory scores; plasma cell scores in PERF+CM versus control were borderline (OR 15.61; *p* = 0.0587).

**Conclusions:**

Perforated dPTFE membranes yielded comparable bone gain to unperforated dPTFE. Adding a collagen membrane over perforated dPTFE did not improve VARA outcomes.

## 1 Introduction

In addition to preventing the ingrowth of connective tissue and maintaining space for regeneration, membranes facilitate appropriate cell colonization and differentiation (Vignoletti and Abrahamsson 2012; Yu et al. 2023). Under this protected area, undifferentiated mesenchymal cells differentiate into osteoblasts, provided the necessary signaling molecules, nutrients, and growth factors are present (Miron et al. 2013). These processes necessitate an adequate blood supply to deliver oxygen and nutrients and a suitable source of mesenchymal cells to ensure optimal conditions for bone regeneration (Kanczler and Oreffo 2008).

Depending on their design and structure, barrier membranes may impede the interaction between the periosteum and the augmented area under the membrane, potentially negatively impacting bone formation (Kostopoulos and Karring 1995; Lin et al. 2014). However, the influence of membrane porosity on bone formation has been a bit controversial. Using a canine model, Gutta and coworkers compared macroporous, microporous, and resorbable meshes with control sites lacking a barrier in an acute defect, where macroporous meshes yielded the highest bone regeneration (Gutta et al. 2009). Inversely, in critical‐size defects, it was found that occlusive devices outperformed porous ones (Polimeni, Albandar, and Wikesjo 2004). Other studies demonstrated that non‐permeable membranes effectively facilitated new bone formation, comparable to or better than permeable membranes, suggesting that membrane permeability is not as critical a factor for guided bone regeneration (Polimeni, Koo, et al. 2004; Schmid et al. 1994). It was also demonstrated that the occlusive value of a barrier membrane might surpass the value of its cell‐permeability (Mardas et al. 2003). A balanced perspective was that membrane porosity significantly affects the rate but not the ultimate quantity of bone neogenesis. Membranes with intermediate porosities balanced optimal tissue integration, stability, and prevention of soft tissue ingrowth (Zellin and Linde 1996).

Finding the right balance between sufficient exclusion of gingival connective tissue ingrowth and adequate permeability to inflammatory cells, mesenchymal cells, growth factors, and nutrients remains a topic of investigation (Jepsen et al. 2019). The literature emphasizes that ideal membranes must be “occlusive with selective permeability” preventing cellular infiltration while allowing diffusion of nutrients, oxygen, and growth factors necessary for osteogenesis (Omar et al. 2019). Direct permeability measurements reveal substantial differences, where collagen membranes were found to have 10‐fold higher initial water permeability than polylactide membranes and 500‐fold higher than ePTFE. These permeability differences correlate with solute diffusion capacity for nutrients like glucose and electrolytes (Zhan et al. 2025). In addition to the enhanced porosity, collagen membranes have proven to play an active bioactive role rather than functioning purely as passive barriers (Turri et al. 2016). Cells recruited into the membrane compartment express signals for bone regeneration, including BMP‐2, FGF‐2, TGF‐β1, and VEGF. The membrane accumulates these growth factors and contains immunoreactive cells that promote bone formation in the underlying defect (Elgali et al. 2017). The value of these growth factors was shown to be especially critical in challenging defects like vertical alveolar ridge augmentation (VARA) (Kammerer et al. 2013).

Thus, the primary objective of the present study is to compare the outcomes of VARA across three configurations: a solid dPTFE membrane, a perforated dPTFE membrane, and a perforated dPTFE membrane with a collagen membrane, using clinical, histomorphometric, and micro‐CT evaluations. As an exploratory secondary objective, differences in inflammatory cell populations, vascular parameters, and immunohistochemical markers of bone metabolic activity were examined to generate hypotheses regarding possible biological responses to membrane perforations.

## 2 Material and Methods

### 2.1 Ethical Considerations and Setting

This experimental in vivo study was carried out at the ATRC Aurigon Toxicological Research Center Ltd. in Budapest (Pálya u. 2., 2120 Dunakeszi, Hungary) with the ethical committee approval of the Pest County government office (Pest Megyei Kormanyhivatal ethical permission No: PE/EA/300‐7/2016) in accordance with the proper institutional and national guidelines for the adequate care of the animals. This investigation was designed and performed following the ARRIVE 2.0 guidelines (Percie du Sert et al. 2020).

### 2.2 Study Design

Study design is illustrated in Figure 1. At T1, following surgical extraction of the mandibular premolars, the same operator (IU) created standardized bilateral ridge defects in all animals. Each defect was approximately 30 mm in the mesiodistal dimension and 8 mm in height. The buccal and lingual crestal plates were reduced to form a flat ridge contour, modeling the atrophic posterior mandibular ridge typically seen in edentulous humans (Figure 1).

**FIGURE 1:**
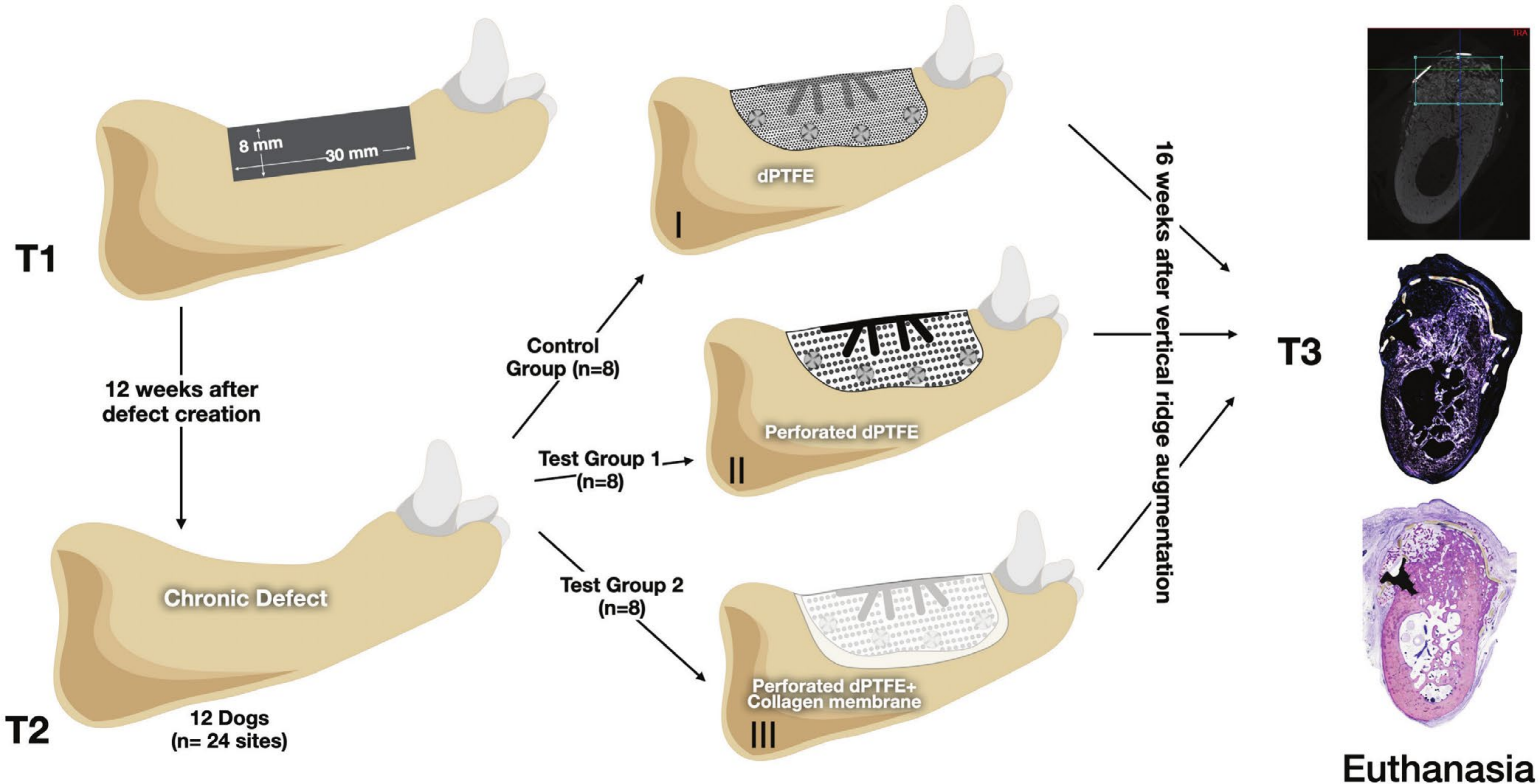
Study design and experimental timeline. Standardized chronic mandibular ridge defects (8 × 30 mm) were created and allowed to heal for 12 weeks (T1–T2) in 12 beagle dogs (24 sites). Sites were then allocated to three interventions: (I) dPTFE membrane (control), (II) perforated dPTFE membrane, or (III) perforated dPTFE membrane + collagen membrane. Animals were euthanized 16 weeks after vertical ridge augmentation (T3), and specimens were harvested for micro‐CT and histologic/histomorphometric analyses.

After 12 weeks of healing (T2), a “chronic supracrestal bone defect experimental model” (Simion et al. 2006) was utilized to evaluate the outcomes of VARA after using anorganic porcine xenograft particles covered with:

- *Control group (NON‐PERF)*: Titanium reinforced dPTFE membrane, or
- *Test group 1 (PERF)*: A modified perforated titanium reinforced dPTFE, or
- *Test group 2 (PERF+CM)*: A modified perforated titanium reinforced dPTFE + collagen membrane.

At T3 (16 weeks of healing post‐VARA), animals were sacrificed, and radiographic and histologic tests were performed.

### 2.3 Randomization, Group Allocation, and Blinding

Sites were randomized evenly to one of three augmentation procedures using a computer‐generated randomization list using a 1:1:1 allocation, with eight sites assigned to each group, carried out in 12 adult beagle dogs (total of 24 sites). The surgeon was blinded to the treatment assignment until the time of membrane placement. The evaluators responsible for assessing the outcomes were not involved in the surgery and did not have access to the treatment allocation information.

### 2.4 Animal Care

Twelve beagle dogs of approximately 32 months of age with a weight ranging between 10 and 20 kg were included in the study. Animals were housed individually during the acclimatization/quarantine period and maintained in purpose‐designed kennels in a 12:12 light/dark cycle at 21°C–22°C in 3‐dog groups during the remaining part of the in‐life phase. The animals received pelleted dog food pre‐softened with water (approximately 300 g of dry food and approximately 500 mL of water added) throughout the study period.

General anesthesia for the surgical procedures was induced by intravenous ketamine, midazolam, and fentanyl. Propofol was administered when needed. Anesthesia was maintained with isoflurane delivered through a tracheal tube and a slow intravenous infusion of ketamine and fentanyl. Local anesthesia was provided using articaine. Postoperative analgesia consisted of a non‐steroidal anti‐inflammatory analgesic, such as meloxicam, and an opioid analgesic, such as tramadol. Infection prevention included daily intramuscular amoxicillin‐clavulanic acid. In addition, surgical wounds were cleaned three times weekly with 0.12% chlorhexidine solution, and the animals received softened food throughout the healing period.

Regarding our criteria for exclusion or early intervention following the intervention and before the endpoint was reached, any signs of systemic distress, persistent infection, or inability to maintain oral intake were monitored daily by veterinary staff.

### 2.5 Study Materials

The bone grafting material used for all groups was anorganic porcine‐derived xenograft (Zcore, Osteogenics Biomedical Inc., Lubbock, TX, USA). However, membrane configuration differed in each group as follows:

*NON‐PERF*: Received a non‐resorbable titanium‐reinforced PTFE membrane (Cytoplast Titanium‐Reinforced Ti‐250, Osteogenics Biomedical Inc., Lubbock, TX, USA).

*PERF*: Received a pre‐perforated (pore size 0.66 mm) titanium‐reinforced PTFE mesh (RPM reinforced PTFE mesh, Osteogenics Biomedical Inc., Lubbock, TX, USA).

*PERF + CM*: Received a pre‐perforated (pore size 0.66 mm) titanium‐reinforced PTFE mesh (RPM reinforced PTFE mesh, Osteogenics Biomedical Inc., Lubbock, TX, USA) that was covered with a porcine‐derived resorbable collagen membrane (Vitala collagen membrane, Osteogenics Biomedical Inc., Lubbock, TX, USA).

### 2.6 Surgical Interventions

#### 2.6.1 Chronic Defect Model Creation

Chronic bone defects measuring 8 mm apicocoronally and 30 mm mesiodistally were created in anesthetized animals through the extraction of the four mandibular premolars and removal of the alveolar process (Simion et al. 2006). Primary wound closure was achieved using a resorbable PGA (polyglycolic acid) suture (Resorba Glycolon, Osteogenics Biomedical, Lubbock, TX, USA), and postoperative analgesia and antibiotics were administered for pain relief and infection prevention.

#### 2.6.2 Vertical Bone Regeneration

After 3 months of healing, full‐thickness mucoperiosteal flaps were elevated. The dimensions of the created bone defects were recorded using a calibrated periodontal probe (UNC‐15). Measurements were taken at standardized reference points from the base of the prepared defect to the most coronal aspect of the recipient alveolar bone using the interproximal bony peak of adjacent teeth as a reference point (Figure 2). During flap elevation, care was taken to preserve the periosteum as much as possible. Cortical perforations were performed to expose the underlying medullary spaces. Bone graft particles were packed into the defect and membranes were trimmed and adapted well to extend beyond the defect margins by 2–3 mm. Membranes were fixed by using 5–7 titanium screws (Pro‐Fix, Osteogenics, Lubbock, USA) in the buccal and lingual bone. Flap advancement was made by a periosteal incision and subsequent blunt coronal extension of the soft tissues to ensure a passive adaptation of the flaps over the wound area. Primary closure was achieved by a double‐layer suturing technique with a non‐absorbable surgical suture (Cytoplast PTFE, Osteogenics Biomedical Inc., Lubbock, TX, USA). After primary closure at the coronal part of the membrane, the flap had periosteum, whereas apically, the underlying connective tissue was in contact with the membrane (Figure 2).

**FIGURE 2:**
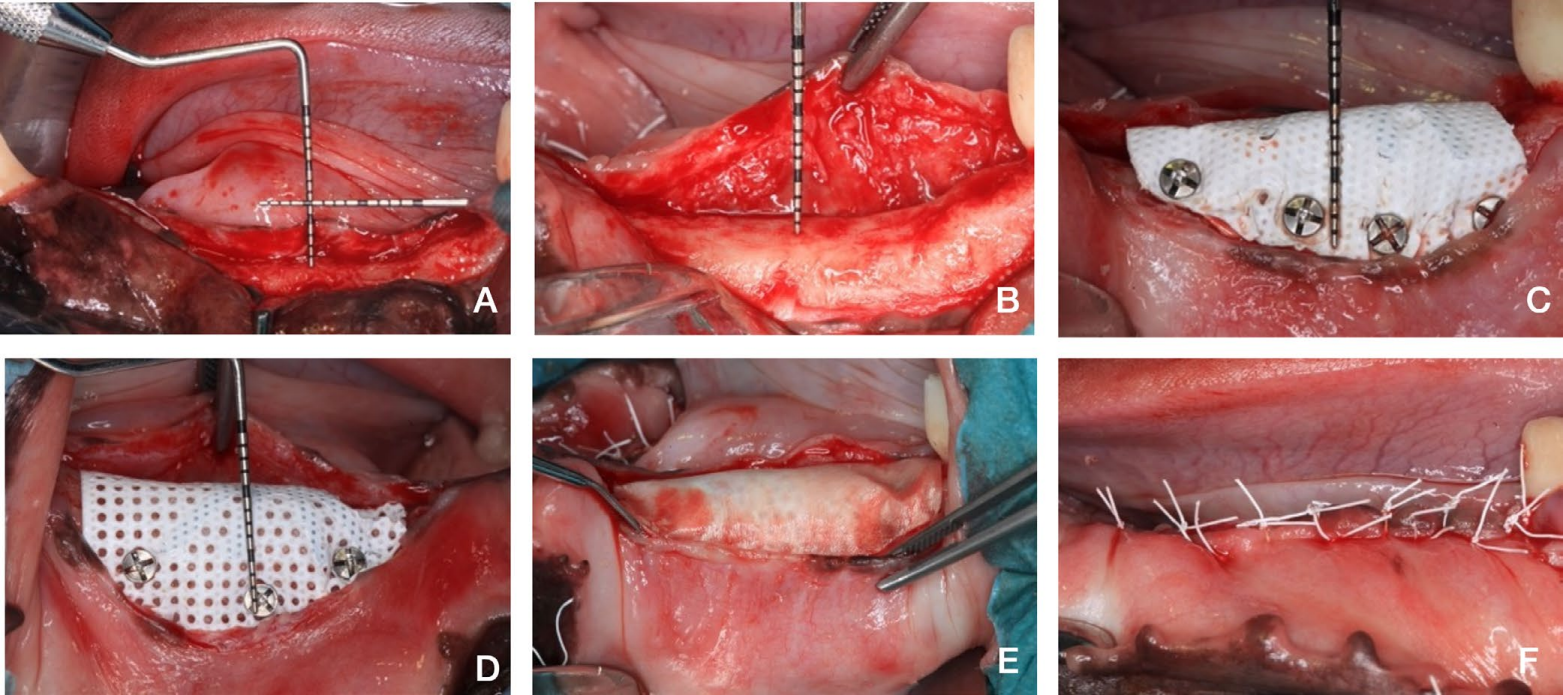
Surgical interventions (A) After 3 months of healing, flaps were elevated and the chronic defects (B) were measured. Each defect was selected for one of the augmentation procedures: PTFE membrane (C), PTFE perforated membrane (D), or PTFE perforated membrane plus collagen membrane (E). All flaps were sutured with a non‐absorbable suture (F).

#### 2.6.3 Post‐Surgical Care

Throughout the entire in‐life phase, general conditions and surgical sites were monitored daily for the initial 2 weeks post‐surgery and weekly thereafter. Animals were provided with softened food, surgical wounds were cleaned thrice weekly using chlorhexidine solution (0.12%), and sutures were removed after 14 days.

#### 2.6.4 Euthanasia and Retrieval of Specimens

After 16 weeks of healing, animals were euthanized with an overdose of sodium pentobarbital (40–60 mg/kg/i.v., Dolethal, Vetoquinol, France). The lower jaws were removed and fixed by immersion in buffered formaldehyde solution and refrigerated.

### 2.7 Specimen Processing

#### 2.7.1 Histological Processing

Tissue blocks containing treated sites were prepared for ground sectioning following Donath and Breuner's technique (Donath and Breuner 1982), with the central area (12 mm) embedded in glycol methacrylate (GMA) resin (Technovit 7200 VLC; Kulzer, Werheim, Germany), and the mesial and distal peripheral regions (approximately 18 mm) embedded in methyl methacrylate (MMA) resin (Technovit 9100; Kulzer, Werheim, Germany) for immunohistochemistry use. Sections were reduced to approximately 50 μm, stained with Lévai‐Laczkó (Lévai et al. 1975), and prepared for each antibody selected (Figure 3).

**FIGURE 3:**
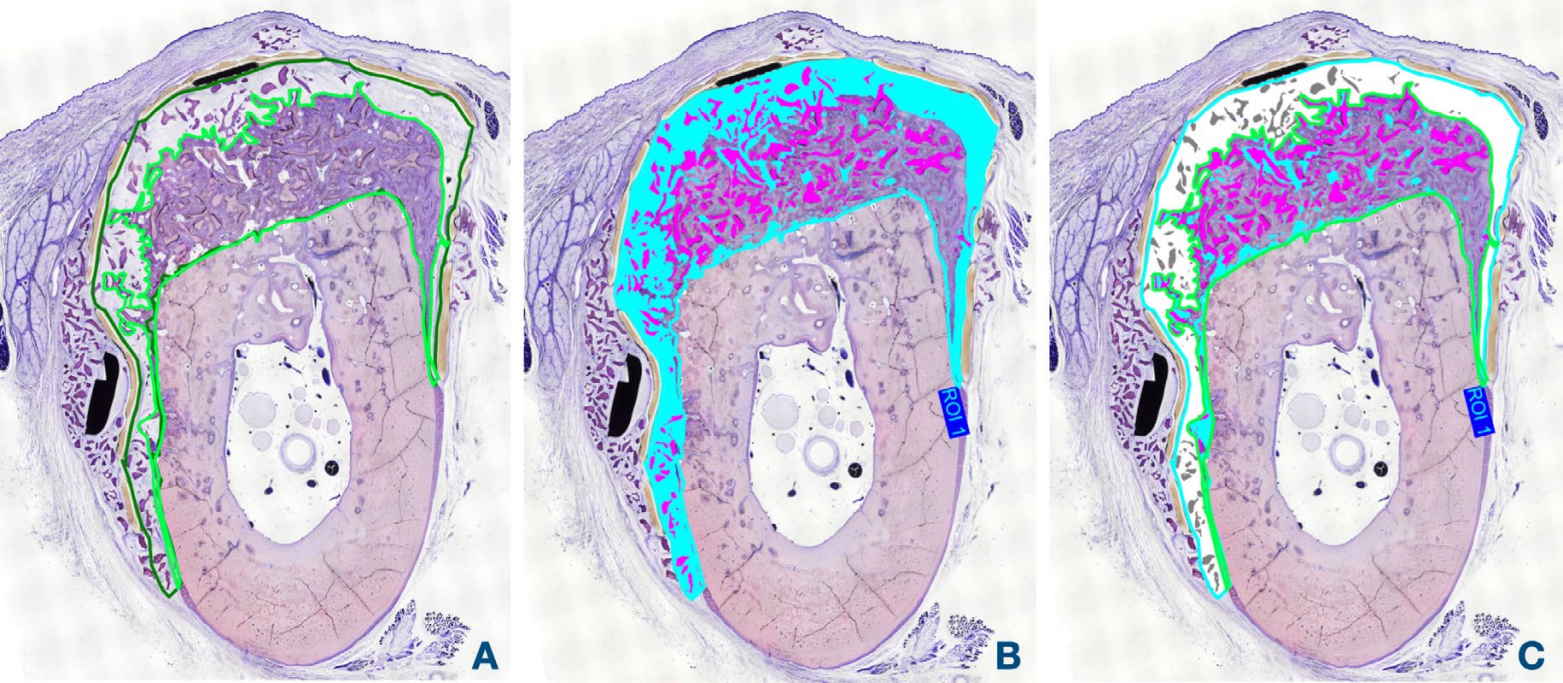
Histomorphometric analysis. Two thin ground sections of each defect were prepared and stained. These sections were acquired and stitched using a motorized microscope. Two regions of interest were defined: (1) “regeneration area” (RA, dark green line), the area between the membrane and the bone defect; (2) “regenerated area” (RegenA, yellow green line) as the part of RA occupied by the newly formed bone (A). Graft particles and soft tissue areas were identified and colored using a digital pen. The percentage of bone, soft tissue, and graft particles was calculated in RA (B) and RegenA (C) areas—magnification x40. Lévai‐Laczkó staining.

#### 2.7.2 Immunohistochemical Labeling

For immunohistochemistry, sections were deplasticized, rehydrated, and treated with proteinase K (Abcam, Cambridge, UK) to unmask antigens. Endogenous peroxidase activity was quenched, and non‐specific binding sites blocked (Agilent, Santa Clara, USA; Vector Lab, Burlingame, USA). Primary mouse monoclonal antibodies included osteocalcin (OCN; Abcam, Cambridge, UK), alkaline phosphatase (ALP), vascular endothelial growth factor (VEGF; Santa Cruz Biotechnology, Dallas, Texas, USA), and tartrate‐resistant acid phosphatase (TRAP; Invitrogen, Thermofisher, Massachusetts, USA). Controls were used to test assay specificity, and antigen–antibody complexes were detected using an anti‐mouse peroxidase‐labeled polymer (EnVision+, DakoCytomation). Color development was achieved with diaminobenzidine (ImmPACT DAB Substrate, Peroxidase; Vector Lab, Burlingame, USA), and the slides were counterstained, dehydrated, and then cover‐slipped.

#### 2.7.3 Micro‐CT Processing

Specimens were scanned before being sectioned using a high‐resolution micro‐CT (Skyscan 1172, Bruker microCT NV, Kontich, Belgium). The X‐ray source was set at 100Kv and 100 μA with a voxel size of 12 μm and an aluminum/copper filter (Al/Cu). The scanning was performed over a 360° rotation, acquiring images every 0.4°, which were later reconstructed using NRecon software (Bruker microCT NV, Kontich, Belgium) using a dedicated algorithm (Feldkamp et al. 1984).

#### 2.7.4 Histological Analysis

Two independent investigators analyzed histological ground sections and were masked to the specific experimental conditions (FM and SF). Digital images were captured with a motorized light microscopy and a digital camera connected to a PC‐based image capture system (BX51, DP71, Olympus Corporation, Japan) and evaluated using image analysis software (CellSens dimensions, Olympus). The following variables were measured in each histological section.

### 2.8 Histomorphometric, Histometric, and Radiographic Measurements

#### 2.8.1 Region of Interest (ROI) Measurements

##### 2.8.1.1 Vertical Bone Regeneration Gain

The maximum height of the regenerated area was calculated from the base of the bone defect to the most coronal mineralized bone underneath the membrane (in continuity with the recipient bone) and was presented as a percentage of the total height of the regeneration (Figure 4A).

**FIGURE 4:**
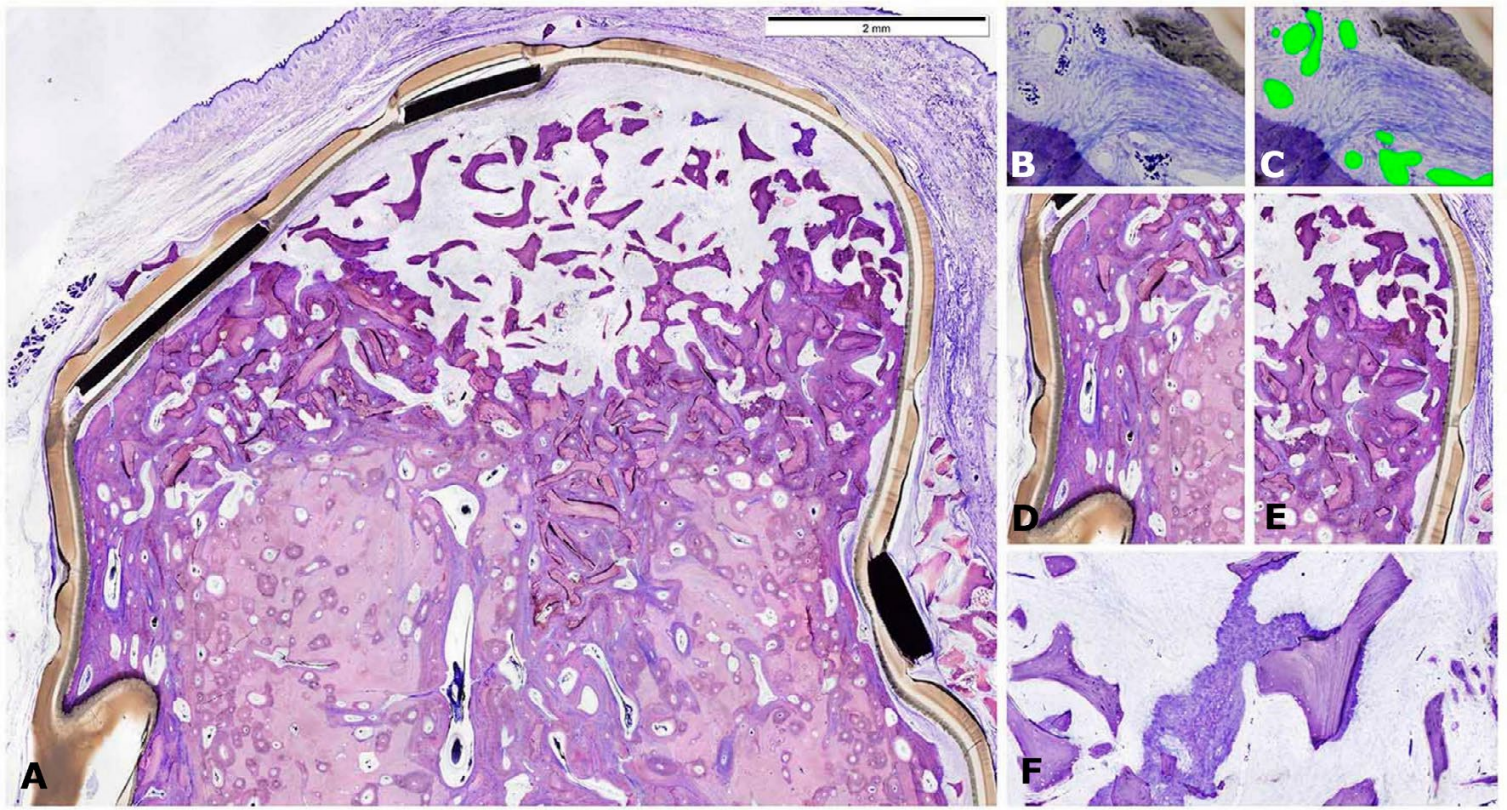
Histomorphometric analysis. Once images were captured (A), two images of each sample at less than 100 μm in the inner surface of the membrane were obtained at a magnification of 20× (B). Blood vessels were colored and quantified as number of vessels and percentage of surface occupied (C). Contact bone‐membrane was determined in the regeneration area (D, E). The perimeter of bone in the limit of the regenerated area was measured (F). Magnification 40× images A, D, and E; magnification 200× images B and C; magnification 100× image F. Lévai‐Laczkó staining.

##### 2.8.1.2 Bone‐To‐Mesh Contact (BMC)

In the regenerated region, the length of the mesh in direct contact with mineralized bone was calculated as a percentage (Figure 4D,E). On the surface of the regenerated bone, collagen fibers were inserted perpendicularly into the newly formed bone resembling new periosteum formation on top of newly corticalized bone (Figure 4B,F).

#### 2.8.2 Semiquantitative Measurements

##### 2.8.2.1 Inflammatory Cells

A semiquantitative evaluation of the number of inflammatory cells (neutrophils, lymphocytes, plasmatic cells, and monocytes) was performed according to the ISO 10993‐6 standard. The following grading scale was used: 0 = absent, 1 = slight (< 5 cells/field), 2 = moderate (5–15 cells), and 3 = marked (> 15 cells).

##### 2.8.2.2 Vascular Structures

To quantify the presence of vessels adjacent to the membrane, two images of each sample of around 430 × 330 μm (camera field) were captured at 20× magnification at the ROI. The images were obtained at less than 100 μm of the membrane, near the pores in perforated dPTFE membrane, or in an equivalent area in the solid dPTFE membrane group. Blood vessels were colored using a pen tablet (Intuos, Wacom, Japan) and image software (Photoshop CS6, Adobe, USA). In each one of the samples, the number of vessels and the percentage of area occupied by the vascular structures in relation to the ROI were determined (mean % area).

#### 2.8.3 Immunohistochemical Analysis

Analysis of markers was performed in three differentiated areas defined as (i) pristine bone, apical to the original defect; (ii) regenerated bone, formed by biomaterial and new bone, and (iii) area under regeneration, in the space between the regenerated bone and the membrane (Orlowska et al. 2026). Three boxes of 1 × 1 mm were created in each one of these areas. Regions of brown color were positive to antibodies were selected manually and automatically counted the percentage of area positive in each one of the boxes (mean% area). The same size and location boxes were used to quantify the collagen content using polarized light.

#### 2.8.4 Micro‐CT Analysis

Images were evaluated with the Data Viewer software (Bruker micro‐CT NV, Kontich, Belgium) once the area of interest was perfectly aligned. Then a rectangular volume of interest (VOI) was selected, avoiding the external margins of the mesh. Data were analyzed with the CTAn software (Bruker micro‐CT NV, Kontich, Belgium) using adaptive local threshold methods for segmenting the images and thus setting the best threshold parameters for the analysis of bone and material. The percentage of bone and the ratio of bone volume to total volume (BV/TV) were measured in the total mesiodistal length of the defect (Figure 5).

**FIGURE 5:**
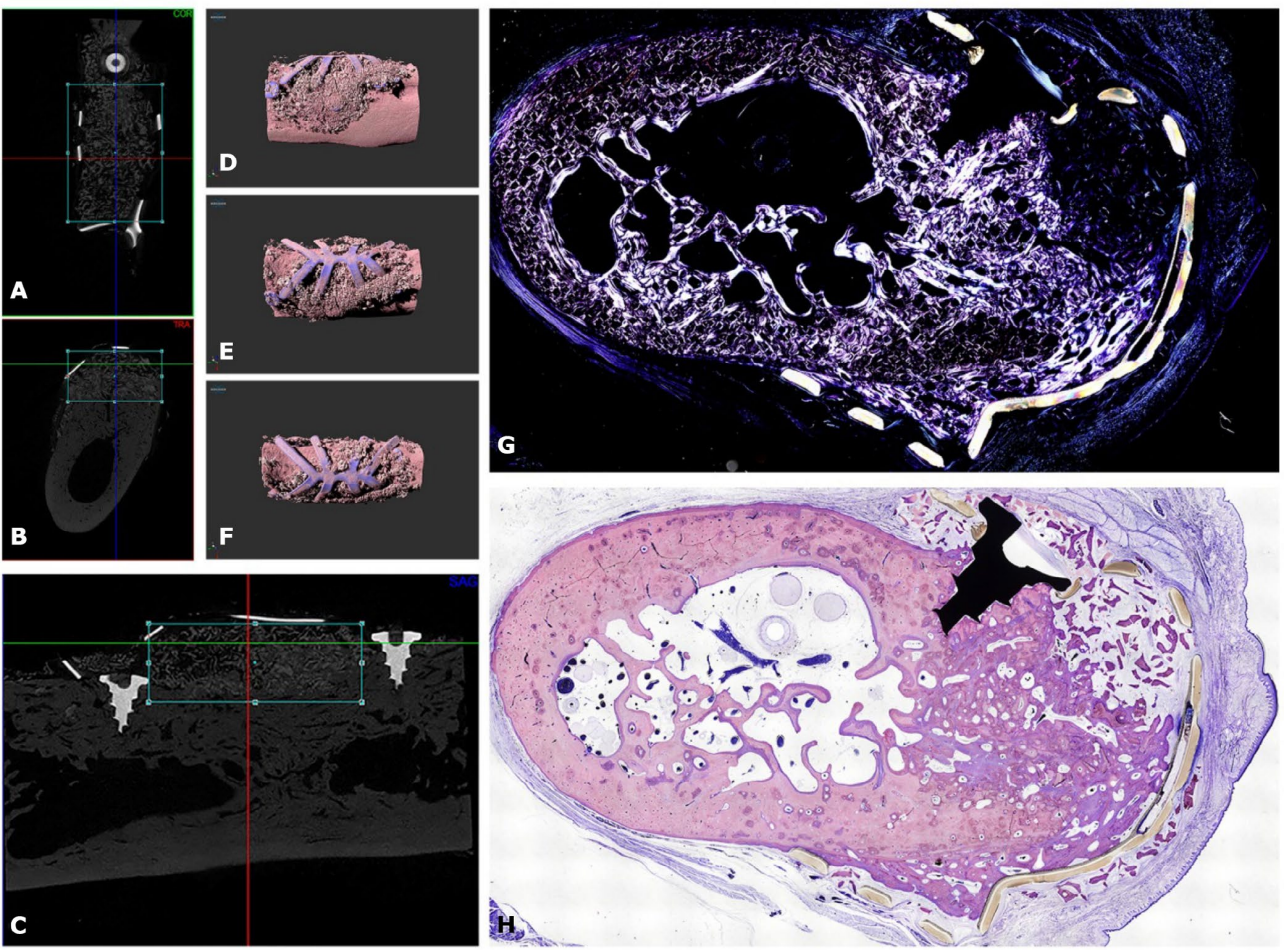
Micro‐CT regions of interest and 3D models (A–F). Original image (H) and the same observed under polarized light (G). An increment in the collagen structure is visible in the areas of new bone compared with the non‐regenerated region.

### 2.9 Statistical Analysis

Data from histological, immunohistochemical, and radiological outcome measurements were analyzed considering the site as the statistical unit of analysis (*n* = 8). Continuous outcomes were summarized by group using the median and interquartile range (25th–75th percentiles). Between‐group differences across the three independent groups were evaluated using the Kruskal–Wallis test (exact *p*‐values). When applicable, post hoc pairwise comparisons were performed using Dunn's multiple‐comparisons procedure with multiplicity‐adjusted *p*‐values. All tests were two‐sided, and statistical significance was set at *α* = 0.05. Analyses were performed using GraphPad Prism (version 10.6.1). Analysis of inflammatory cells was done using ordinal logistic regression.

## 3 Results

### 3.1 Main Clinical Observations

The general condition of the dogs was good during the entire in‐life phase, as demonstrated by their eating and drinking ad libitum. From 24 treated sites, four sites were excluded due to post‐surgical complications, including dehiscence and progressive wound exposure. Some cases were managed with local wound care and surgical debridement followed by tension‐free primary closure, while others evolved from early soft‐tissue thinning to partial and, in some instances, complete exposure over subsequent weeks. In total, site allocation/group at the time of histological analysis was 7:8:5 for the NON‐PERF, PERF, and PERF+CM groups respectively.

### 3.2 Main Histological Observations

All groups demonstrated comparable histologic outcomes. Xenograft particle displacement was common, and the contour of the regenerated region was frequently altered, most likely due to animal biting. Within regenerated sites, biomaterial granules were incorporated into woven and lamellar bone, yielding an almost complete osseous organization of the original defects. The membrane interface showed either direct bone contact or separation by a thin connective tissue layer in which collagen fibers were typically parallel to the membrane, with occasional vertical fibers extending into new bone. Polarized light microscopy demonstrated increased collagen fiber content in regenerated areas compared with pristine lamellar bone, whereas non‐regenerated regions consisted of dispersed biomaterial particles within collagen fibers with relatively reduced collagen fiber density.

Membranes were generally encapsulated internally and externally by a collagen‐rich, pauci‐cellular connective tissue with fibers oriented parallel to the barrier. Collagen enrichment in remodeling/new bone formation zones was similar across modalities. In the PERF group, membrane pores contained highly vascularized connective tissue, with dense collagen bundles traversing pores and anchoring to the membrane and sometimes to newly formed bone; poor membrane adaptation was associated with fiber redirection toward the internal aspect, while well‐adapted areas more often showed direct membrane–bone contact regardless of perforations.

### 3.3 Histological Outcomes

In total, 40 ground sections were utilized for histological evaluation. Histomorphometric and histometric results are presented in Table 1.

**TABLE 1:** Histomorphometric outcomes following VARA.

	Non‐Perf median (IQR)	Perf median (IQR)	Perf + CM median (IQR)	*p* (Kruskal–Wallis)
Area of new bone, soft tissue and residual graft particles assessments (%)				
New bone (%)	63.55 (61.17–71.63)	64.68 (57.32–68.66)	56.83 (54.21–70.30)	0.5047
Soft tissue (%)	16.09 (12.38–17.32)	16.34 (10.70–19.98)	17.95 (16.25–22.23)	0.2994
Residual graft particles (%)	22.01 (15.67–26.13)	23.02 (15.36–25.04)	22.19 (12.94–25.59)	0.9689
BV/TV (%)	0.8391 (0.8268–0.8762)	0.8366 (0.8002–0.8930)	0.8205 (0.7777–0.8375)	0.2994
Vertical bone regeneration				
Linear vertical gain (mm)	2.330 (2.100–3.310)	2.865 (2.058–3.218)	2.740 (2.475–3.685)	0.7119
Vertical gain area (%)	67.07 (35.23–85.13)	67.45 (48.65–68.72)	55.11 (41.26–75.54)	0.8495
Bone‐to‐mesh contact				
Bone‐to‐mesh contact (%)	65.29 (55.07–82.86)	59.52 (52.19–66.11)	65.93 (41.36–69.21)	0.7268
Collagen fibers infiltrating in newly formed bone				
Length of area with collagen insertion (mm)	3.22 (2.63–3.35)	4.33 (2.68–4.70)	11.95 (4.40–15.33)	**0.0467** *
	Dunn's multiple comparisons test		Non‐Perf vs. Perf	> 0.9999
			Non‐Perf vs. Perf + CM	**0.0526**
			Perf vs. Perf + CM	0.2185
Vascular structures				
Number of vessels (n)	6.00 (5.00–8.00)	8.50 (5.88–9.38)	8.00 (5.25–8.25)	0.1699
Area with vascular structures (%)	4.76 (2.77–7.22)	8.37 (5.95–11.10)	4.40 (2.19–6.17)	**0.0522**
	Dunn's multiple comparisons test		Non‐Perf vs. Perf	0.228
			Non‐Perf vs. Perf + CM	0.8097

*Note:* Vertical bone regeneration was measured from apical base of defect to most coronal bone under the membrane; bone‐to‐mesh contact (BMC) expressed as percentage of mesh length in direct contact with mineralized bone; bundle bone–like collagen fiber length measured along the coronal crest where collagen fibers were incorporated vertically into the newly formed tissue (mm). Group comparisons were performed using Kruskal–Wallis test with Dunn's post hoc multiple comparisons when applicable. Bold values are significant.

* *p* < 0.05 indicates statistical significance.

#### 3.3.1 Area of New Bone, Soft Tissue, and Residual Graft Particles Assessments

The proportion of newly formed bone, soft tissue, and residual graft particles within the regenerated area was comparable among groups. Median (Q1–Q3) new bone was 63.55 (61.17–71.63) in the NON‐PERF group, 64.68 (57.32–68.66) in PERF, and 56.83 (54.21–70.30) in PERF + CM (Kruskal–Wallis, *p* = 0.5047). Differences in soft tissue and residual graft were not significant (*p* = 0.2994 and 0.9689, respectively) (Figures 6 and 7).

**FIGURE 6:**
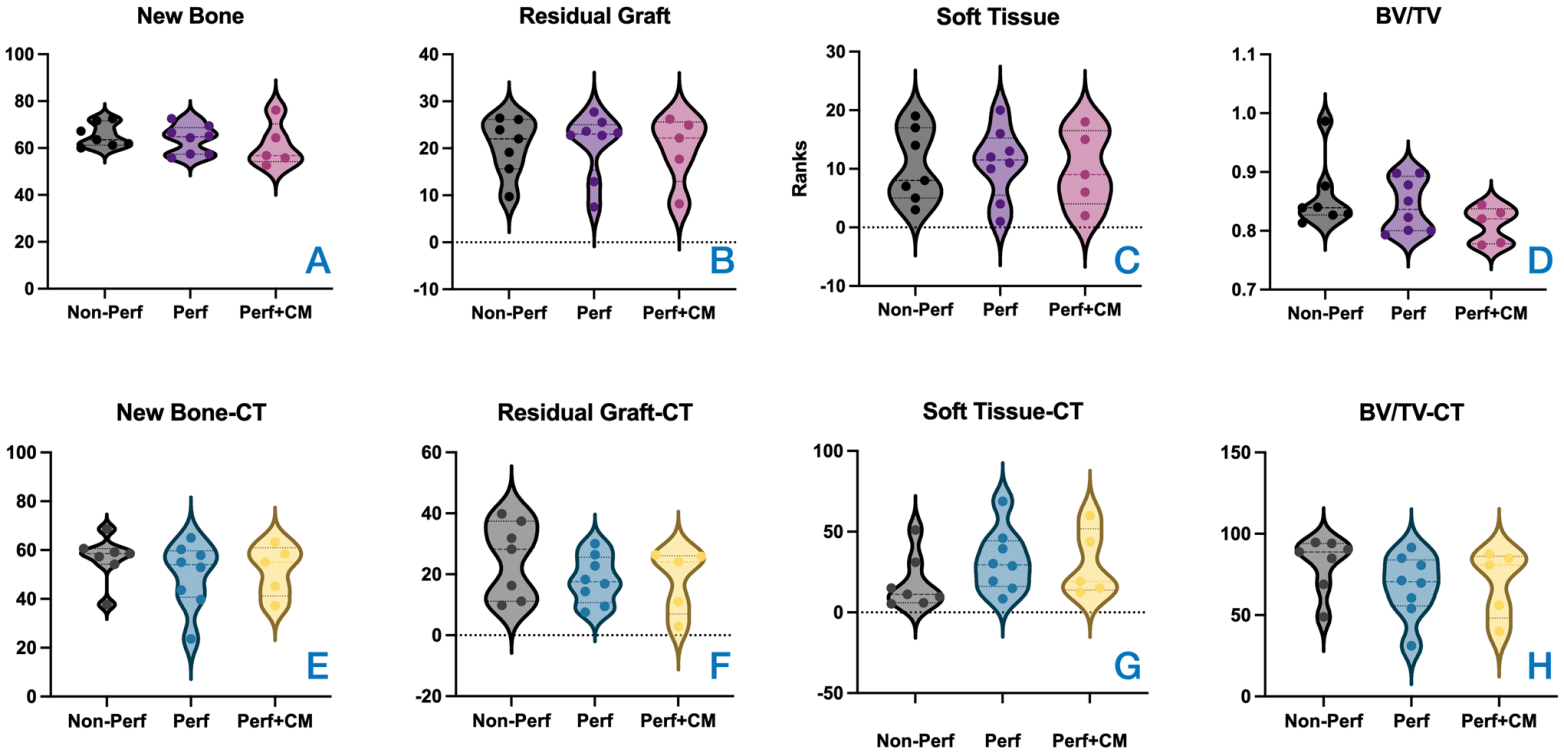
Micro‐CT and histomorphometric outcomes by treatment group. Violin plots depict the distribution of (A) new bone, (B) residual graft, (C) soft tissue, and (D) BV/TV, and their corresponding CT‐based measures (E–H) across Non‐Perf, Perf, and Perf + CM groups. Individual data points are overlaid; dashed lines indicate the reference level used for visualization.

**FIGURE 7:**
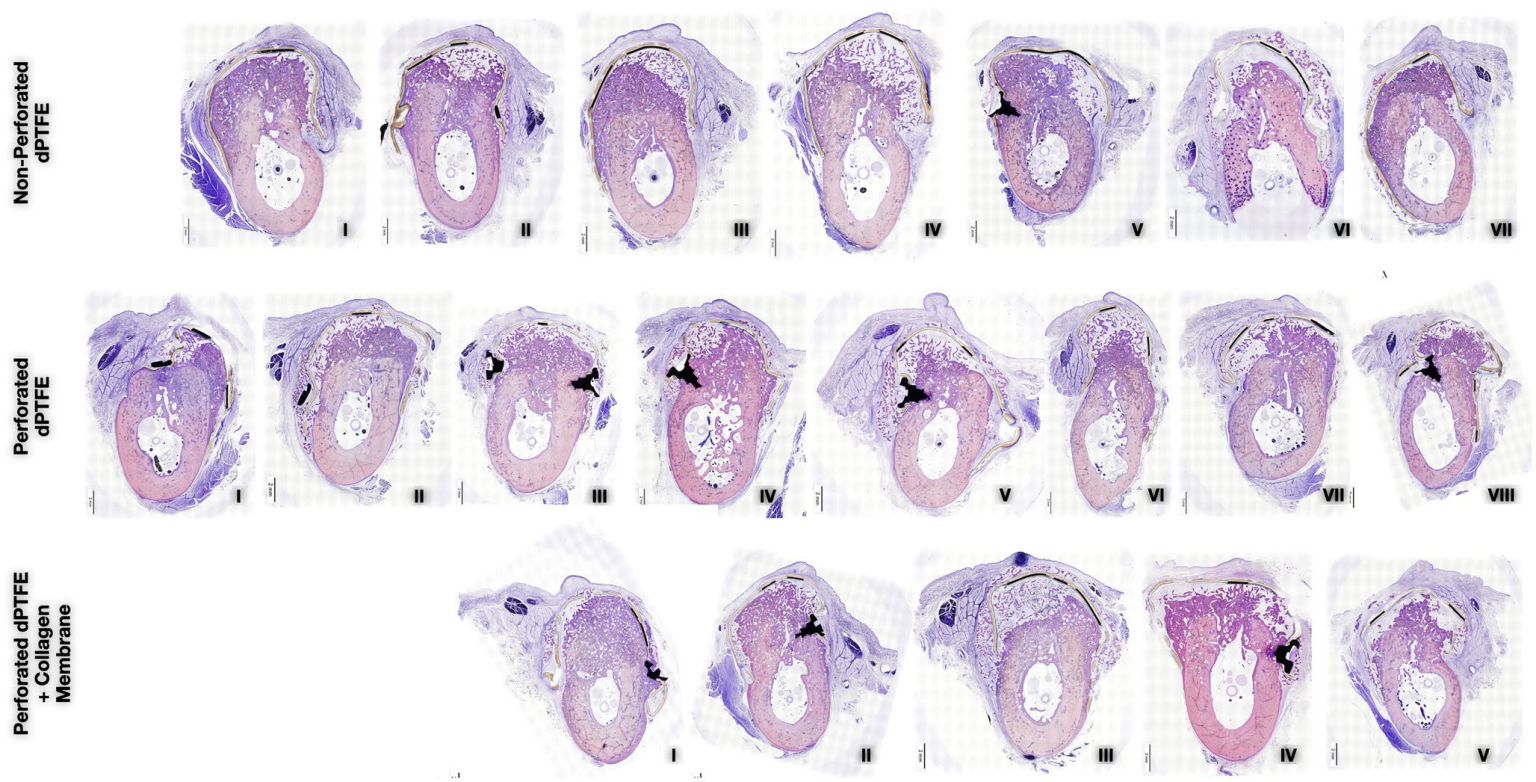
Representative histologic overview of the regenerated defects by treatment group. Low‐magnification ground sections from sites treated with non‐perforated dPTFE (control), perforated dPTFE, and perforated dPTFE plus collagen membrane are shown. Images illustrate the overall architecture of the augmented region and the relationship between the barrier membrane, regenerated tissues, and residual graft particles across individual specimens.

#### 3.3.2 Vertical Bone Regeneration

The coronal extension of the regenerated area (mm) was similar across groups, with medians of 2.330, 2.865, and 2.740 for the NON‐PERF, PERF, and PERF + CM groups (Kruskal–Wallis, *p* = 0.7119). The percentage of gained regenerated area (%) was 67.07 (35.23–85.13), 67.45 (48.65–68.72), and 55.11 (41.26–75.54), respectively (Kruskal–Wallis, *p* = 0.8495) (Figure 8).

**FIGURE 8:**
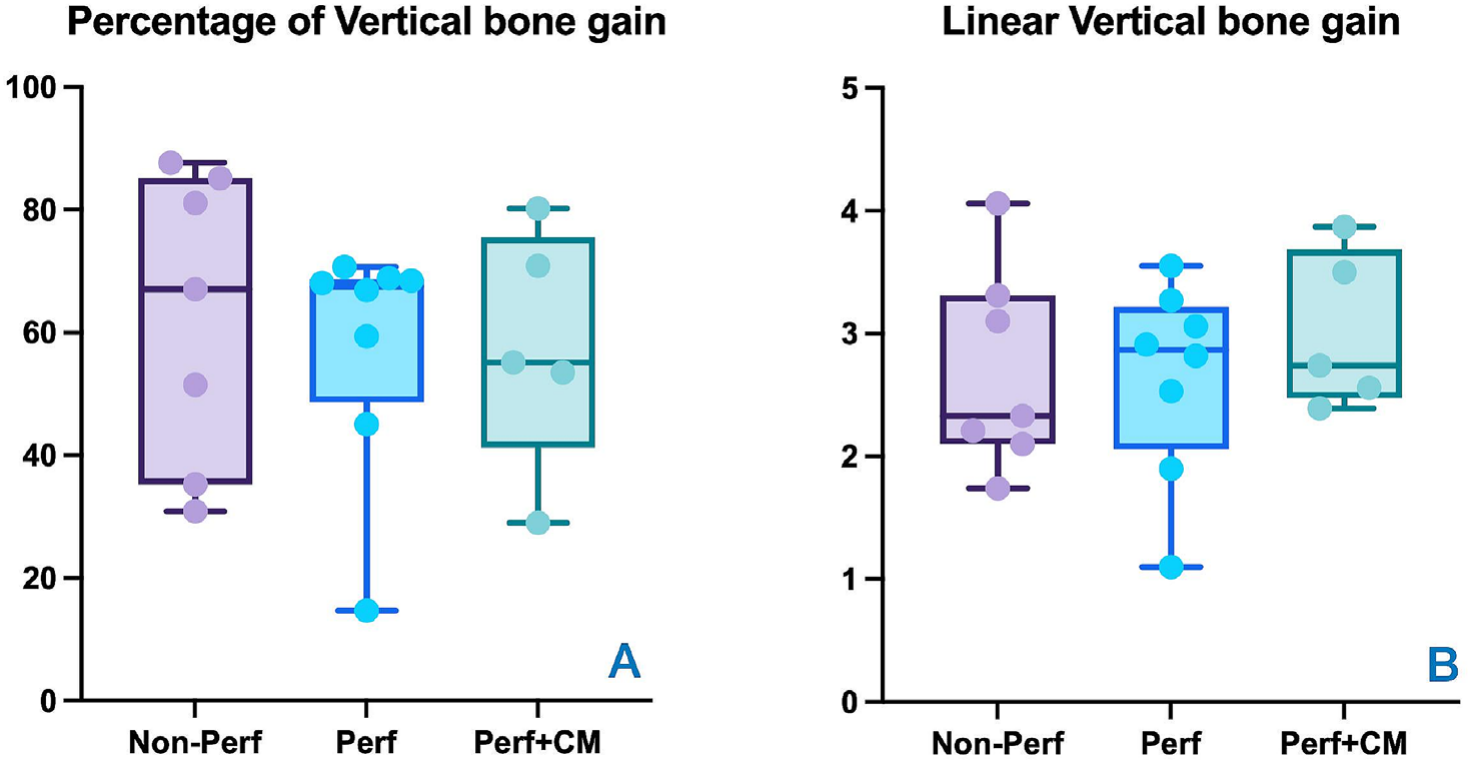
Vertical ridge gain by treatment group. Box‐and‐whisker plots show (A) percentage of vertical bone gain and (B) linear vertical bone gain (mm) for Non‐Perf, Perf, and Perf + CM groups. Individual data points are overlaid.

#### 3.3.3 Bone‐To‐Mesh Contact

BMC was comparable among groups (*p* = 0.7268). The length of connective tissue inserting into newly formed bone in contact with the membranes (mm) showed an overall group difference (*p* = 0.0467), with medians of 3.22 (2.63–3.35), 4.33 (2.68–4.70), and 11.95 (4.40–15.33) for NON‐PERF, PERF, and PERF + CM groups, respectively. Post hoc Dunn's multiple comparisons did not identify a statistically significant pairwise difference after adjustment (NON‐PERF vs. PERF: *p* > 0.9999; and PERF vs. PERF + CM: *p* = 0.2185), with a marginal significance between NON‐PERF vs. PERF + CM (*p* = 0.0526).

#### 3.3.4 Inflammatory Cells

Ordinal logistic regression did not identify statistically significant differences in semiquantitative inflammatory cell scores (0–3) between groups (Table 2). Only for PERF + CM vs. NON‐PERF, a marginally significant odds ratios (OR) for higher scores were found for plasmatic cells, OR 15.61 (0.90–269.58; *p* = 0.0587).

**TABLE 2:** Ordinal logistic regression for inflammatory cell infiltration scores (0–3): Odds ratios (95% CI) and *p*‐values for Perf and Perf + CM vs Non‐Perf.

Outcome (0–3)	Comparison	SE	*z*	*p*	OR	OR_CI95_low	OR_CI95_high
Neutrophils	Perf vs. Non‐Perf	0.981967	0.39736	0.691102	1.477267	0.215571905	10.12339
	Perf + CM vs. Non‐Perf	1.215625	1.258387	0.208252	4.616917	0.426177895	50.01648
Lymphocytes	Perf vs. Non‐Perf	1.353217	−0.89939	0.368447	0.296098	0.02087154	4.200661
	Perf + CM vs. Non‐Perf	1.241832	0.441634	0.658754	1.730544	0.15174479	19.73565
Plasmatic cells	Perf vs. Non‐Perf	1.339023	0.355069	0.722538	1.608731	0.11659615	22.19641
	Perf + CM vs. Non‐Perf	1.453515	1.890568	**0.058682**	15.610911	0.904005266	269.5787
Monocytes	Perf vs. Non‐Perf	1.175589	−0.2224	0.824	0.76993	0.076872294	7.711397
	Perf + CM vs. Non‐Perf	1.254361	1.441281	0.149505	6.097545	0.521700528	71.26705

*Note:* A semi quantitative evaluation of the number of inflammatory cells neutrophils, lymphocytes, plasmatic cells, and monocytes was performed according to the ISO 10993‐6 standard. Bold values are significant.

#### 3.3.5 Vascular Structures

The number of vessels was similar among groups (*p* = 0.1699). The percentage (%) of the ROI occupied by vascular structures was marginally significant (*p* = 0.0522). When Dunn's multiple comparisons versus NON‐PERF were examined, no statistically significant differences were detected (PERF vs. NON‐PERF: *p* = 0.228; PERF + CM vs. NON‐PERF: *p* = 0.8097).

### 3.4 Immunohistochemical Outcomes

OCN activity was more pronounced in both the areas under regeneration and of regenerated bone in the PERF group, as compared to NON‐PERF and PERF + CM groups, and these differences were statistically significant (*p* < 0.05). Similarly, ALP activity was more pronounced in PERF compared with the other groups, but the differences were statistically significant only in the regenerated bone area (*p* < 0.05). VEGF expression was similar in all treatment groups and locations. TRAP activity was significantly increased in PERF + CM in the area under regeneration (*p* < 0.05) (Table S1).

### 3.5 Micro‐CT Outcomes

In total, 20 images resulting from micro‐CT processing were analyzed. No statistically significant differences were detected among groups for any micro‐CT outcome (Kruskal–Wallis: graft particles *p* = 0.3974, soft tissue *p* = 0.2739, bone *p* = 0.5774, BV/TV *p* = 0.2739) (Table 3).

**TABLE 3:** Micro‐CT outcomes by group.

Variable	Non‐Perf median (IQR)	Perf median (IQR)	Perf + CM median (IQR)	*p* (Kruskal–Wallis)
Graft particles (%)	28.20 (11.17–37.42)	17.59 (10.70–25.54)	24.11 (6.955–26.10)	0.3974
Soft tissue (%)	11.16 (5.920–31.03)	29.48 (16.03–44.35)	19.20 (13.82–51.85)	0.2739
New bone (%)	58.51 (54.22–60.64)	54.03 (40.67–59.71)	55.00 (41.21–60.94)	0.5774
Bone volume/tissue volume (%)	88.84 (68.97–94.08)	70.52 (55.65–83.97)	80.80 (48.16–86.19)	0.2739

*Note:* The radiographic measurements were performed considering mean graft particles, soft tissues, new bone, and bone volume/tissue volume (BV/TV). All data is presented in percentage (%) of area fraction of region of interest (ROI).

## 4 Discussion

In this canine chronic defect model of VARA, outcomes after use of a non‐perforated titanium‐reinforced dPTFE membrane, a perforated dPTFE membrane, or a perforated dPTFE membrane covered with a collagen membrane were broadly comparable. Histomorphometry and micro‐CT demonstrated similar distributions of newly formed bone, soft tissue, residual graft, and BV/TV across groups, and vertical ridge gain was not different among treatments. Bone‐to‐mesh contact was likewise comparable. A single histologic parameter—bundle bone–like connective tissue insertion length along the coronal crest—showed an overall group difference (Kruskal–Wallis *p* = 0.0467), with higher values in the perforated dPTFE plus collagen group; however, adjusted post hoc testing did not confirm a statistically significant pairwise difference. Semiquantitative inflammatory cell scores analyzed by ordinal logistic regression did not demonstrate significant between‐group differences but plasma cell scores in the collagen groups compared to the control group were borderline (OR 15.61; *p* = 0.0587). Consequently, considering the primary outcome variables, the study's hypothesis was rejected.

On the other hand, when considering the secondary outcome variables, slight differences have been observed. A more pronounced metabolic bone and vascular activity have been observed when utilizing the perforated PTFE membrane. This enhanced cell proliferation results in the presence of angiogenic and osteogenic cells, thereby providing a crucial supply of blood, oxygen, and nutrients to the tissues (Abrahamsson et al. 2012; Urban, Farkasdi, et al. 2025). A higher degree of mineralization, osteoblastic activity, vascularization, and maturation of regenerated bone was observed when using a perforated membrane. Other studies inferred the periosteum's importance in providing adequate nutrition and essential growth factors for osteogenesis (Kostopoulos and Karring 1994, 1995; Linde et al. 1993). However, these observations should be interpreted only as hypothesis‐generating since they did not translate into statistically significant improvements in new bone formation, vertical bone gain, BV/TV, or other primary histomorphometric and micro‐CT outcomes. Therefore, the present findings do not support a conclusion of superior biological performance or enhanced bone maturation in the perforated membrane group. These findings align with classical studies by Simion et al., who used a similar experimental model employing e‐PTFE membranes for vertical bone regeneration (Simion et al. 2007).

Immunohistochemical analysis was performed to evaluate the concentration of cells positive to OCN, ALP, VEGF, and TRAP. ALP was found in higher concentrations in the regenerated area in the PERF group, demonstrating higher osteoblastic activity. Similarly, OCN was observed in higher concentrations in two of the investigated areas (regenerated and under regeneration areas), demonstrating higher mineralization in the PERF group. However, these marker‐level differences cannot establish accelerated bone maturation or improved regenerative performance in the absence of corresponding significant differences in the primary bone formation outcomes. On the other hand, TRAP was more elevated in the PERF + CM group, demonstrating higher osteoclastic activity. These findings, together, may be in part explained by the resorbable feature of the covering collagen membrane in the PERF + CM group and the role inflammatory cells play in membrane degradation and consequent bone resorption and fibrous tissue formation.

It should be taken into consideration that the dPTFE membrane is an occlusive barrier but still presented a micro‐porosity that allowed the access of molecules and tissue fluids. In the Perf + CM group, the membrane presented a slow resorption rate that may have hampered the influence of cells on osteogenesis. Similarly, the absence of differences in PERF + CM could be attributed to the collagen membrane covering, which hindered periosteum function, however, this mechanism was not directly tested in the present study. Recently a randomized clinical trial comparing dPTFE alone versus dPTFE with an added collagen membrane additionally demonstrated that adding a collagen membrane may help prevent soft tissue ingrowth into the regenerative space (Urban, Serroni, et al. 2025).

While some studies suggest that augmented pore size has a positive effect (Gutta et al. 2009), others demonstrate the benefits of an occlusive dense barrier membrane (Polimeni, Albandar, and Wikesjo 2004) for bone regeneration. The optimal membrane porosity and permeability remain undefined. Clinically, the observed higher vascularization and bone metabolic activity may indicate faster, more mature bone formation for early implant placement stages. A similar titanium‐reinforced perforated PTFE mesh showed better vertical bone gain and improved vascularization in a recent human case series (Urban et al. 2021). These results imply that absolute cell exclusion might not be essential for guided bone regeneration rather, it could contribute substantially to bone formation in challenging defects addressed in this investigation. Nevertheless, the hypothesis still needs to be further corroborated, and randomized prospective clinical trials are needed to compare vertical bone augmentation results using various space‐making frameworks (Fiorellini et al. 2026; Saleh et al. 2026).

Both CM and dPTFE membranes appear to perform bioactive roles in VARA not merely passive barriers (Elgali et al. 2017). In vivo and human data indicate that PTFE membranes can upregulate osteogenic activity in the underlying defect, promoting earlier osteoprogenitor differentiation and increasing osteocalcin expression (Matsuzaka et al. 2001; Tanaka et al. 2007). In periodontal regeneration, dPTFE was associated with increased osteopontin and ALP and concomitant upregulation of growth‐factor, inflammatory, and remodeling pathways (e.g., FGF‐2, IL‐6/IL‐1, RANKL/OPG, MMP‐2/MMP‐9) versus flap surgery alone (Amar et al. 1997; Lima et al. 2008). By contrast, naturally derived collagen membranes have been shown to modulate defect healing through coordinated effects on bone formation/remodeling (e.g., osteocalcin, CTR/CatK/RANKL) and by “tuning” inflammatory and recruitment mediators (e.g., TNF‐α dynamics; CXCR4 and MCP‐1), consistent with a role in orchestrating osteogenic and osteoclastic phases (Omar et al. 2019; Turri et al. 2016). Collagen membranes may function as a biologically active compartment by supporting infiltration of membrane‐associated cells with osteogenic marker expression (e.g., ALP, OPN, osteocalcin) and demonstrating intra‐membrane immunoreactivity for pro‐osteogenic growth factors (e.g., FGF‐2, BMP‐2), with reported correlations between molecular activity within the membrane and the underlying defect tissue (Taguchi et al. 2005). In the present study, adding a collagen membrane over the perforated dPTFE did not translate into improved radiographic or histomorphometric outcomes, despite the reported bioactive potential of collagen membranes. One possible explanation is that, in this model, the non‐resorbable space‐maintaining dPTFE compartment and standardized grafted environment may have limited the detectable incremental effect of the collagen membrane. Future studies should consider a design that is aimed to isolate the specific cellular pathways responsible for the observed secondary marker patterns.

Preclinical in vivo models are crucial before human trials; however, they don't perfectly replicate human oral conditions (Elrefaei et al. 2025). Understanding the limitations of these models is essential for effective research and accurate interpretation of preclinical findings (Stavropoulos et al. 2015). Another notable limitation of this preclinical study was the absence of sample size calculation, as the experiment adhered to the 3Rs principle to minimize animal use. The bone healing dynamics following defect creation can lead to significant variations among baseline bone defects. While only anorganic porcine‐derived graft particles were used, differences in membrane adaptation and fixation may arise in these supracrestal bone defects among treatment groups (Chacon et al. 2024). To minimize discrepancies and ensure standardization during bone defect creation, all surgeries were performed by a single, highly experienced surgeon (IU).

## 5 Conclusion

Similar new bone formation has been observed histologically and radiographically when comparing the three regenerative strategies. Adding a collagen membrane over the perforated dPTFE did not improve the VARA histomorphometric or radiographic outcomes.

Secondary exploratory analyses suggested possible improvements in vascular and bone‐remodeling marker patterns with the perforated membrane; however, these findings were not accompanied by superior histomorphometric or micro‐CT outcomes.

## Author Contributions

**Mauricio G. Araujo:** writing – review and editing. **Fabio Vignoletti:** writing – review and editing, methodology, investigation, project administration. **Istvan A. Urban:** conceptualization, investigation, funding acquisition, supervision, methodology. **Gabor Varga:** investigation, writing – review and editing. **Sándor Farkasdi:** writing – review and editing, writing – original draft, data curation, investigation, formal analysis. **Muhammad H. A. Saleh:** writing – original draft, project administration, visualization. **Monica Lopez:** writing – review and editing, visualization, investigation. **Fernando Muñoz:** writing – review and editing, visualization, investigation.

## Funding

This study was partially supported by the Osteogenics Biomaterials.

## Conflicts of Interest

The authors declare no conflicts of interest.

## Supporting information


**Table S1:** Immunohistochemical and histochemical marker expression (OCN, ALP, VEGF, TRAP) in pristine bone, area under regeneration, and regenerated bone: mean (SD) across groups. Alkaline phosphatase (ALP) activity evaluates osteoblastic activity and bone formation. The osteocalcin (OCN) activity evaluates mineralization level. The vascular endothelial growth factor (VEGF) evaluates vascularization. Tartrate resistant acid phosphatase (TRAP) evaluates osteoclasts activity. Analysis of markers was performed in three differentiated regions: (i) pristine bone; (ii) regenerated bone and (iii) area under regeneration. All data are presented in percentage (%) of area fraction of region of interest (ROI). Kruskal–Wallis Test (Nonparametric ANOVA), Dunn's Multiple Comparisons Test.
**Figure S1:** Alkalin Phosphatase (ALP) is located in regions of osteoid formation (a). Osteocalcin (OCN), produced by osteoblasts works as bridge between collagen fibers and the mineral matrix (b). Tartrate‐resistant acid phosphatase (TRAP) positive cells were found around graft particles non‐osseointegrated (c). Vascular endothelial growth factor (VEGF) positive around a particle surrounded by pristine bone, indication an increment in the blood vessel formation (d). All antibodies were thresholded in three regions: non‐regenerated area, regenerated area, and pristine bone far from the defect. Bars show magnification.
**Figure S2:** Semiquantitative inflammatory cell scores by treatment group. Violin plots show the distribution of 0–3 scores for (A) lymphocytes, (B) monocytes, (C) neutrophils, and (D) plasma cells across Non‐Perf, Perf, and Perf + CM groups. Individual data points are overlaid, and dashed lines denote the score reference levels.
**Figure S3:** Vascularization adjacent to the membrane by treatment group. Box‐and‐whisker plots show (A) percentage of ROI occupied by vascular structures and (B) number of vessels for Non‐Perf, Perf, and Perf + CM groups. Individual data points are overlaid.

## Data Availability

The data that support the findings of this study are available from the corresponding author upon reasonable request.
