# Supplementary material for: Effect of Membrane Perforations With and Without Collagen Membrane Coverage in Vertical Ridge Augmentation: An Experimental Pilot In Vivo Study
Source: Clin Oral Implants Res. 2026 Jul 19;37(10):1158–71. doi: 10.1111/clr.70157 (PMC13644542; doi:10.1111/clr.70157)
Supplement: Supplementary file 1 [file CLR-37-1158-s001.docx]

|  | Non-Perf Mean (SD) | Perf Mean (SD) | Perf+CM Mean (SD) |
| --- | --- | --- | --- |
| OCN |  |  |  |
| Pristine bone | 5,92 ± 7,03 | 17,53 ± 10,40 | 18,25 ± 14,38 |
| Area under regeneration | 8,55 ± 4,92 | **17,05 ± 5,94*** | 12,51 ± 11,80 |
| Regenerated bone | 2,05 ± 1,16 | **7,10 ± 3,92*** | 1,31 ± 1,57 |
| ALP |  |  |  |
| Pristine bone | 0,05 ± 0,05 | 0,07 ±0,30 | 0,12 ± 0,18 |
| Area under regeneration | 0,31 ± 0,23 | 0,30 ± 0,61 | 0,95 ± 0,57 |
| Regenerated bone | 1,73 ± 0,92 | **4,59 ± 3,49*** | 0,61 ± 0,38 |
| VEGF |  |  |  |
| Pristine bone | 1,56 ± 2,84 | 0,85 ± 1,13 | 1,82 ± 1,77 |
| Area under regeneration | 1,61 ± 2,54 | 0,51 ± 0,53 | 1,32 ± 2,18 |
| Regenerated bone | 0,72 ± 0,66 | 0,56 ± 0,50 | 0,34 ± 0,22 |
| TRAP |  |  |  |
| Pristine bone | 0,02 ± 0,02 | 0,05 ± 0,08 | 0,02 ± 0,01 |
| Area under regeneration | 0,43 ± 0,50 | 0,40 ± 0,21 | **2,41 ± 2,28*** |
| Regenerated bone | 0,06 ± 0,08 | 0.05 ± 0,05 | 0,17 ± 0,16 |

**Supplemental Table S1. Immunohistochemical and histochemical marker expression (OCN, ALP, VEGF, TRAP) in pristine bone, area under regeneration, and regenerated bone: mean (SD) across groups**. Alkaline phosphatase (ALP) activity evaluates osteoblastic activity and bone formation. The osteocalcin (OCN) activity evaluates mineralization level. The vascular endothelial growth factor (VEGF) evaluates vascularization. Tartrate resistant acid phosphatase (TRAP) evaluates osteoclasts activity. Analysis of markers was performed in three differentiated regions: i) pristine bone; ii) regenerated bone and iii) area under regeneration. All data are presented in percentage (%) of area fraction of region of interest (ROI). Kruskal-Wallis Test (Nonparametric ANOVA), Dunn's Multiple Comparisons Test

*	Statistically significant difference (p< 0.05) between control and PERF+CM vs PERF

** Statistically significant difference (p< 0.05) between control and PERF vs test

**(Supplementary Figure S1).**

**Supplementary Figure S1** Alkalin Phosphatase (ALP) is located in regions of osteoid formation (a). Osteocalcin (OCN), produced by osteoblasts works as bridge between collagen fibers and the mineral matrix (b). Tartrate-resistant acid phosphatase (TRAP) positive cells were found around graft particles non-osseointegrated (c). Vascular endothelial growth factor (VEGF) positive around a particle surrounded by pristine bone, indication an increment in the blood vessel formation (d). All antibodies were thresholded in three regions: non-regenerated area, regenerated area, and pristine bone far from the defect. Bars show magnification.

**Supplementary Figure S2.**

**Supplemental Figure S2. Semiquantitative inflammatory cell scores by treatment group.** Violin plots show the distribution of 0–3 scores for (A) lymphocytes, (B) monocytes, (C) neutrophils, and (D) plasma cells across Non-Perf, Perf, and Perf+CM groups. Individual data points are overlaid, and dashed lines denote the score reference levels.

**Supplemental Figure S3. Vascularization adjacent to the membrane by treatment group.** Box-and-whisker plots show (A) percentage of ROI occupied by vascular structures and (B) number of vessels for Non-Perf, Perf, and Perf+CM groups. Individual data points are overlaid.
